# Pulmonary inflammatory leiomyosarcoma represents a potential diagnostic pitfall of DNA methylation-based classification of sarcomas: a case report

**DOI:** 10.1186/s12890-023-02624-z

**Published:** 2023-09-04

**Authors:** Takahiro Shibayama, Kaishi Satomi, Ryota Tanaka, Akihiko Yoshida, Kiyotaka Nagahama, Akimasa Hayashi, Takashi Hibiya, Kazuharu Suda, Masachika Fujiwara, Junji Shibahara

**Affiliations:** 1 Department of Pathology, Kyorin University School of Medicine, 6-20-2 Shinkawa, Mitaka, Tokyo, 181-8611 Japan; 2 Department of General Thoracic Surgery, Kyorin University School of Medicine, Tokyo, Japan; 3 Department of Diagnostic Pathology, National Cancer Center Hospital, Tokyo, Japan; 4 Rare Cancer Center, National Cancer Center Hospital, Tokyo, Japan

**Keywords:** Sarcoma, Pulmonary, Inflammatory leiomyosarcoma, DNA methylation

## Abstract

**Background:**

Pulmonary inflammatory leiomyosarcoma (PILMS) is a rare type of myogenic tumor with prominent lymphohistiocytic infiltration. Despite their histological similarities, PILMS is immunohistochemically and genetically distinct from soft tissue inflammatory leiomyosarcoma, and its clinicopathological picture including DNA methylome data remains still unknown.

**Case presentation:**

Here we present a case of PILMS in an 18-year-old male who underwent lobectomy. As reported previously, the current case demonstrated spindle myoid cell proliferation with smooth muscle differentiation within a prominent lymphohistiocytic infiltration and a diploid genome with a *MUC3A* gene alteration. DNA methylation analysis predicted this case to be an “inflammatory myofibroblastic tumor” (IMT) according to the Deutsches Krebsforschungszentrum (DKFZ) classifier. The data was analyzed by t-distributed stochastic neighbor embedding, which plotted the case tumor in the vicinity of IMT, however, there were no IMT histological features. These discordant results could be due to background non-neoplastic inflammatory cells.

**Conclusions:**

As the DNA methylation classification of PILMS might be a potential diagnostic pitfall, an integrative histological and genetic approach is required for its accurate diagnosis.

## Background

Inflammatory leiomyosarcoma (ILMS) was first reported in 1995 [1] and introduced as a rare malignant neoplasm, characterized by smooth muscle differentiation with a prominent lymphohistiocytic infiltration and genomic near-haploidization, in the 5th edition of the World Health Organization (WHO) classification of soft tissue and bone tumors [2]. As ILMS shares clinicopathologic and genetic features with the recently identified “histiocyte-rich rhabdomyoblastic tumor (HRRMT)” [3–5], these two subtypes were proposed to be reclassified as a unified entity termed “inflammatory rhabdomyoblastic tumor (IRMT)” [6, 7].

Kao and colleagues [8] demonstrated that pulmonary inflammatory leiomyosarcoma (PILMS) is immunohistochemically and genetically distinct from IRMT, despite their shared histological features. Whole exome and transcriptomic sequencing revealed a diploid karyotype and no convincing skeletal muscle differentiation among four cases of PILMS in young to middle-aged males. Recently, DNA methylation-based classification has been introduced for the pathological diagnosis of central nervous system tumors and sarcomas [9]. However, a DNA methylation profile of PILMS has not been reported thus far.

Herein, we report a rare PILMS case that was successfully analyzed using the Illumina Infinium Methylation EPIC BeadChip array v1.0 (Illumina, San Diego, California, USA) and a fresh frozen specimen, to assess whether the Deutsches Krebsforschungszentrum (DKFZ) classifier could be utilized for this emerging tumor type or not. The DNA methylome signature of PILMS was also visualized using t-distributed stochastic neighbor embedding (tSNE).

## Case presentation

An 18 year-old Japanese male who complained of chest discomfort for two months was admitted to the Kyorin University Hospital. Chest computed tomography revealed a well-demarcated mass in the right upper lung with atelectasis. A bronchoscopy procedure showed complete bronchial obstruction due to external tumor compression. The patient underwent lobectomy, and no relapse was noted for one year after surgery.

**Fig. 1:**
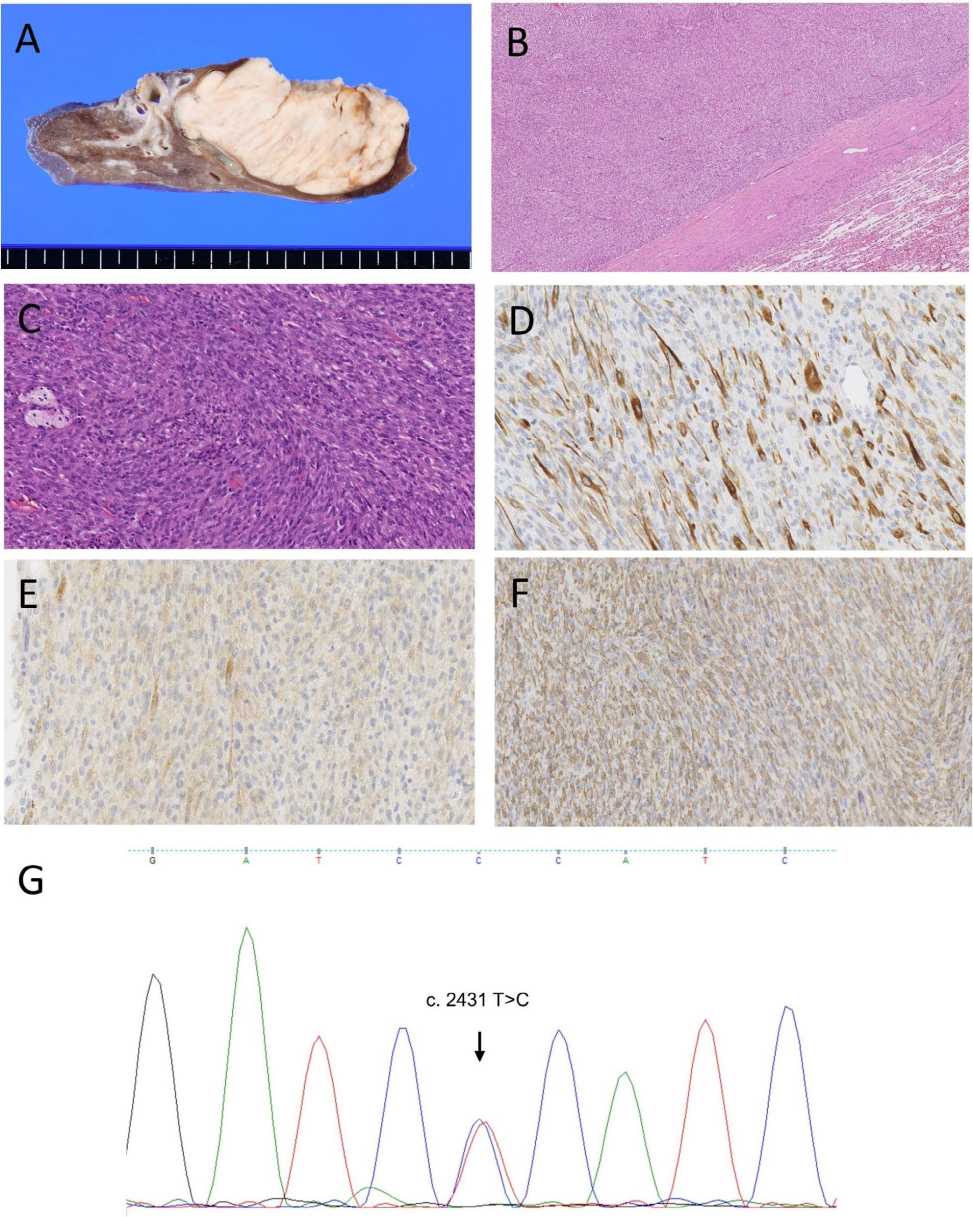
Pathological and genomic features of pulmonary inflammatory leiomyosarcoma. **a** Macroscopically, the tumor was observed as a well-demarcated firm white mass. **b** Histologically, the tumor was focally encapsulated by a fibrous capsule at low magnification. **c** Tumor cells had eosinophilic cytoplasm and blunt-ended nuclei arranged in intersecting fascicles with scattered foamy histiocytes at high magnification. **d** Tumor cells were focally positive for desmin. **e** A few neoplastic cells were weakly positive for h-caldesmon. **f** Numerous histiocytic infiltrations were highlighted upon CD163 immunohistochemical analysis. **e**, Sanger sequencing revealed the *MUC3A* gene alteration p. S811P (c. 2431 T > C).

Macroscopically, the tumor was a well-defined white-colored firm mass, of 7.3 cm in diameter (Fig. 1a). Histological examination using hematoxylin and eosin staining showed a well-circumscribed and focally encapsulated nodule in the peripheral lung parenchyma compressed bronchi. The peripheral sclerotic change was focally identified (Fig. 1b). Relatively uniform spindle cells with eosinophilic cytoplasm and blunt-ended nuclei were observed to be proliferating in the intersecting fascicles (Fig. 1c). Mild to moderate nuclear atypia without pleomorphism was identified. The mitotic count was 6/mm^2^, and necrosis was not observed. Scattered multinucleated giant cells and aggregates of foamy histiocytes were observed as characteristic features. Immunohistochemically, the tumor cells were focally positive for desmin (Fig. 1d) and heavy isoform of caldesmon (h-caldesmon, clone: h-CD, Dako, Glostrup, Denmark) (Fig. 1e) and negative for smooth muscle actin (SMA) and rhabdomyoblastic markers, including Myogenin and MyoD1. The presence of cytokeratin (AE1/AE3), S100 protein, MDM2, CDK4 ALK, ROS1, and panTRK was not detected. A prominent histiocytic infiltration was highlighted upon CD163 immunostaining (Fig. 1f).

Break-apart fluorescence in situ hybridization (FISH) for SS18 (18q11.2), one of the most frequently used probes in routine diagnostic practice, confirmed the diploidy of the tumor and demonstrated no split pattern with two distinct fused (red/green) signals.

PILMS was previously characterized by mutations in the *MUC3A* gene [8]. To confirm this, Sanger sequencing of the *MUC3A* gene was carried out after PCR amplification with a pair of primers (F: TGTAAAACGACGGCCAGTCCACCACCACCAAGACC, R: CAGGAAACAGCTATGACTGATGCCCACGGATGA). The template DNA was extracted from a surgically resected fresh frozen specimen. A single nucleotide variant of the *MUC3A* gene [p. S811P (c. 2431 T > C), Fig. 1g] was confirmed.

**Fig. 2:**
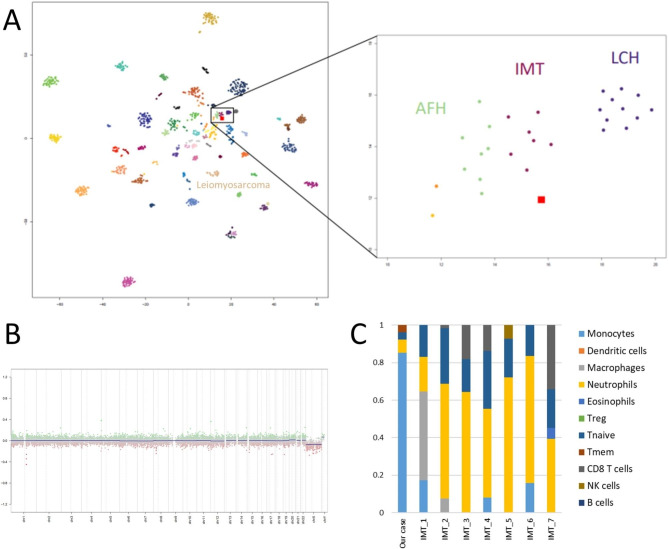
DNA methylation profile of pulmonary inflammatory leiomyosarcoma. **a** Using t-distributed stochastic neighbor embedding, our case (red square) was plotted in the vicinity of inflammatory myofibroblastic tumors (IMTs). The color code used for 62 reference tumors and three control DNA methylation classes is the same as that described in the article by Koelsche et al. [9]; AFH: angiomatoid fibrous histiocytoma and LCH: Langerhans cell histiocytosis. The methylation class leiomyosarcoma is indicated in the left panel. **b** A copy number plot derived from DNA methylome data demonstrates a relatively silent chromosomal copy number status. **c** The immune cell profile of PILMS shows a larger fraction of monocytes and a smaller fraction of neutrophils as compared to IMT.

Genome-wide DNA methylome data was also successfully obtained using the fresh frozen specimen. The DKFZ classifier (sarcoma classifier v12.2) predicted the case to be of “methylation class inflammatory myofibroblastic tumor (IMT)” with a high calibrated score of 0.91. To visualize the DNA methylome signature, the 428,230 probes selected after filtering were included in a tSNE plot using the Rtsne package (version 0.15). In the tSNE plots, our case was plotted in the vicinity of, but did not fall into, IMT among the reference samples consisting of 1,077 sarcomas (GSE140686) [9] (Fig. 2a). The relative copy number alterations based on the DNA methylation array demonstrated an overall flat profile (Fig. 2b). The bulk DNA methylome profile was compared to seven IMT cases selected from the reference dataset using the MethylResolver package downloaded from GitHub (https://github.com/darneson/MethylResolver) [10], and our case had a larger fraction of monocytes and a smaller fraction of neutrophils when compared to IMT (Fig. 2c).

## Discussion and conclusion

Recent studies propose IRMT [6, 7], to include ILMS [3, 4] and HRRMT [5] since they express rhabdomyoblastic markers including Myogenin, MyoD1, and PAX7. The immunoreacrtivity of h-caldesmon, which is considered one of the most specific markers of smooth muscle differentiation when compared to desmin or SMA, was also absent. The present PILMS case showed no evidence of skeletal muscle differentiation, similar to the four cases reported by Kao et al. [8]. In addition, the *MUC3A* single nucleotide variant supported the diagnosis of PILMS [8]. The significance of the variants is not listed in the COSMIC (https://cancer.sanger.ac.uk/cosmic) or ClinVar (https://www.ncbi.nlm.nih.gov/clinvar/) databases, and the pathogenic impact of this alteration is unknown (score is not available) according to PolyPhen-2 (http://genetics.bwh.harvard.edu/pph2/). Although the DNA methylation array can only evaluate relative copy number alterations compared to control samples, the overall flat profile of our case suggests a diploid karyotype owing to two copies of the SS18 locus determined by FISH. Furthermore, a copy number alteration of chromosomes 5, 20, and 22, which is frequently observed in IRMT, was not observed. Therefore, we have confirmed that PILMS is independent and distinct from IRMT, which is characterized by rhabdomyoblastic differentiation and a near-haploid genome [6, 7].

Interestingly, DNA array-based classification indicated this case as an IMT. Despite the DKFZ classifier prediction, PILMS differs from IMT in terms of morphological, immunophenotypical, and genomic aspects. First, IMT is composed of amphophilic bipolar-shaped myofibroblastic spindle cell proliferation, accompanied by lymphoplasmacytic infiltration. In contrast, PILMS is characterized by eosinophilic myoid cell proliferation within numerous histiocytic infiltrations. Second, IMT shows more frequent positivity for SMA than desmin, while PILMS demonstrates frequent desmin expression [8]. Third, IMT harbors definitive fusion genes, including *ALK* and *ROS1*. As the calibrated score of the DKFZ classifier is robust regardless of tumor purity [9, 11], our unexpected result was thought to be due to the influence of background non-neoplastic lymphohistiocytic infiltration in PILMS.

Interestingly, two tumor entities with non-neoplastic inflammatory infiltration, namely angiomatoid fibrous histiocytoma and Langerhans cell histiocytosis, were also depicted close to our case on the tSNE plot. The positional relation of those tumors might suggest the presence of the inflammatory cell rich superfamily although the tSNE plot preserved limited global structure. Furthermore, as highlighted by morphological findings, our case had a larger fraction of monocytes and a smaller fraction of neutrophils when compared to IMT. Thus, unlike the DKFZ classifier estimation, neither the tSNE plot nor the immune cell repertoire was consistent with the case being an IMT.

One limitation of the immune cell repertoire analysis based on bulk DNA methylome data is that it showed a predominant fraction of neutrophils in six out of seven reference IMT samples, even though the typical histology of IMT indicates non-neoplastic lymphoplasmacytic infiltration. There are two possible explanations for this discrepancy. First, the epithelioid variant of IMT is documented to exhibit predominant neutrophil infiltration [12]. Although the histological details of the seven reference IMT samples are not publicly available, the potential inclusion of epithelioid IMT should be considered. Second, the characteristics of the algorithm may lead to overestimation of the neutrophil fraction for reasons that are currently undetermined.

In conclusion, we have herein reported a very rare case of PILMS and obtained its DNA methylome signature. Both the tSNE plot and the inflammatory cell repertoire of the specimen were consistent with the morphological distinction of PILMS. Although DNA methylome-based classification may have caveats, DNA methylome analysis of large numbers of PILMS cases is a promising ancillary tool for the further characterization of unique biological features of tumor cells. Integrated usage of morphology and molecular diagnostic approaches is required to assess this emerging tumor type.

## Data Availability

The datasets generated during and/or analyzed during the current study are available from the corresponding author on reasonable request.
